# Debilitating Manifestation of a Disease with Multiple Names: A Severe Case of Sclerosing Mesenteritis

**DOI:** 10.1155/2021/6629424

**Published:** 2021-02-08

**Authors:** Valeriia Klymenko, Reba Varughese, Katherine A Woolley, Joseph M Wetherell, Haritha Mopuru, Edward Mensah, Imran Siddiqui

**Affiliations:** Department of Medicine, St. Vincent's Medical Center, Bridgeport, CT, USA

## Abstract

Sclerosing mesenteritis (SM) is a rare inflammatory condition with unknown etiology that affects the mesenteric adipose tissue. We present a case of a 49-year-old male with severe abdominal pain who underwent abdominal biopsy confirming the presence of adipose inflammation and necrosis. The diagnosis of SM was made, and the patient was treated with prednisone and tamoxifen. As this condition is rare, there are no standard guidelines for management. This case aims to outline a possible treatment plan.

## 1. Introduction

Sclerosing mesenteritis (SM) is a rare, idiopathic disorder involving inflammation and fibrosis of the mesenteric adipose tissue [1]. SM is noted to be poorly understood and difficult to classify as it has a wide range of definitions and terminology. It was first described in 1924 and named retractile mesenteritis [2]. The term sclerosing mesenteritis is used to encompass many similar conditions including mesenteric panniculitis, mesenteric fibrosis, retractile mesenteritis, mesenteric lipodystrophy, and misty mesentery which are all distinguished histologically [2]. A pathologic predominance of fibrosis as opposed to inflammation is found in mesenteric fibrosis, whereas inflammation predominance indicates mesenteric panniculitis, with fat necrosis indicating mesenteric lipodystrophy [3]. With an estimated prevalence ranging from >1%–1.26%, this rare condition may lead to a wide array of nonspecific symptoms such as abdominal pain, weight loss, change in bowel habits, nausea and vomiting, anorexia, and fever [1, 2]. On a review of 92 cases of SM, 51 patients (51%) had a normal abdominal exam, 22 (24%) had tenderness, 14 (15%) had an abdominal mass, and 13 (14%) had chylous ascites [4]. The nondescript physical exam findings with a majority of patients having a normal physical exam make this condition difficult to recognize. The etiology remains largely unknown with cases suggesting association with trauma, autoimmune conditions, infection, and malignancy [1]. Computed tomography (CT) is the diagnostic imaging of choice, while biopsy is utilized to rule out malignancy. SM is often an incidental finding on abdominal imaging, and since this condition is extremely rare, there are insufficient data about the clinical course and therefore no standard guidelines for management. We present a case of SM treated with prednisone and tamoxifen to contribute to the existing knowledge base on SM.

## 2. Case Presentation

A 49-year-old male with a past medical history of hypertension, hyperlipidemia, posttraumatic stress disorder, and depression presented to the emergency department with complaints of diffuse abdominal pain, diarrhea, weight loss, night sweats, and subjective fever.

One month prior to admission, the patient suddenly developed diffuse, sharp abdominal pain, which was associated with nonbloody diarrhea, nausea, and night sweats; it was aggravated by eating. History was notable for a 40 lb unintentional weight loss prior to the onset of symptoms. The patient's surgical history was insignificant. Family history was notable for lymphoma in a first-degree relative and an uncle who died from colon cancer at the age of 62.

On examination, vital signs were unremarkable. Physical examination revealed diffuse abdominal tenderness without rebound tenderness or guarding. Rectal exam was normal, and the stool occult was negative for blood. Laboratory studies were notable for leukocytosis 12.2 × 10^3^/mcL with left-sided shift, erythrocyte sedimentation rate 20 mm/hr, and mild transaminitis; electrolytes, creatinine, lipase, C-reactive protein, lactic acid, lithium levels, ANA, and rheumatoid factor were all within normal limits. Stool examination revealed no ova or parasites, and C. diff toxin and culture were also negative.

**Figure 1:**
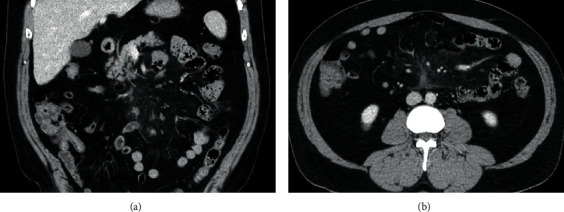
CT abdomen w/contrast: there is moderate “misty” infiltration of the root of the small bowel mesentery (arrows), where an increased number of subcentimeter normal-size mesenteric lymph nodes are also identified. (a) Coronal view. (b) Axial view.

**Figure 2:**
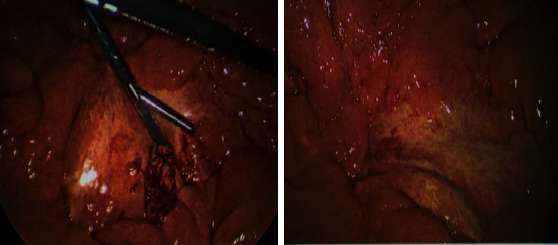
Mosaic white stranding of the mesentery in the proximal aspect in the areas involved on CT imaging. The bowel was hyperemic but obviously viable.

**Figure 3:**
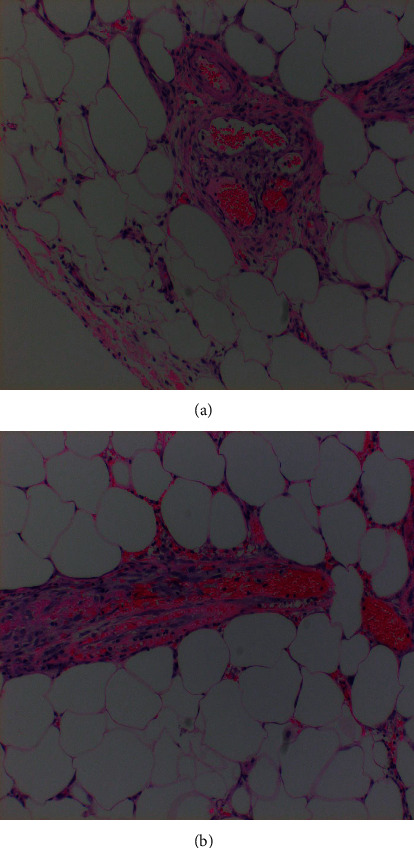
Histopathologic findings of (a) Soft tissue, small bowel mesentery, biopsy: mature adipose tissue with patchy chronic inflammation and fat necrosis; (b) soft tissue, epiploic appendage, biopsy: mature adipose tissue with focal acute and chronic inflammation. Focal small vessel vasculitis and foci of microthrombi.

CT scan of abdomen and pelvis with contrast was done demonstrating hazy infiltration with subcentimeter lymph nodes, slightly more prominent in the right abdomen, which was concerning for sclerosing mesenteritis and mild splenomegaly (Figure 1). The hospital course was complicated by severe pain and vomiting requiring intravenous opiates. Diagnostic laparoscopy was pursued which revealed mosaic white stranding of the proximal mesentery in the areas involved (Figure 2). Biopsy of small bowel mesentery showed mature adipose tissue with patchy chronic inflammation and fat necrosis; thus, the diagnosis of SM was established (Figure 3). The patient was initially treated with tamoxifen 10 mg BID and prednisone 40 mg with no improvement within 3 months of therapy. This patient was not able to tolerate an oral diet, suffering abdominal pain and watery diarrhea after each meal. Overall, he had 60 lbs of weight loss. In the next phase of treatment following steroid/tamoxifen resistance, the patient was started on total parenteral nutrition (TPN), adalimumab 80 mg subcutaneously every 14 days and pentoxifylline 400 mg PO TID, while steroids were tapered off in an appropriate manner. Four months after treating the patient with adalimumab/pentoxifylline therapy, he started feeling better, and he was taken off TPN, able to tolerate 1 meal/day, and maintained weight.

Meanwhile, the patient was still experiencing night sweats periodically. Since SM is associated with various malignancies, including GI adenocarcinoma, the patient underwent an extensive workup. Esophagoduodenoscopy/colonoscopy was done and did not reveal any significant abnormality. PET scan was also performed and did not demonstrate any lymphadenopathy or abnormal FDG activity within the mesentery. Liver, gallbladder, adrenal glands, spleen, and pancreas had normal appearance as well.

## 3. Discussion

Sclerosing mesenteritis is a diagnosis of exclusion, as conditions such as lymphoma, carcinoid tumor, and carcinomatosis could present in a similar fashion. On CT, the most specific features for SM are the “fat ring sign” and tumor pseudocapsule surrounding the inflammation. SM could also present as a soft tissue homogeneous/heterogeneous mass, sometimes with calcifications from fat necrosis or as an increased attenuation of mesenteric fat without mass, which is called “misty mesentery” [1]. As this condition is extremely rare and multiple nomenclature were used in the past, we have limited understanding of its etiology, pathophysiology, and clinical course. It has been postulated to be associated with a history of abdominal surgery; autoimmune conditions such as lupus and rheumatoid arthritis; infectious agents such as tuberculosis, cryptococcus, schistosomiasis, HIV, and cholera; medications including paroxetine and pergolide; and malignancy [1]. However, it remains controversial as multiple case-control studies have brought these associations into questions. Two studies failed to show any association between SM and prior abdominal trauma, surgery, comorbidities such as diabetes mellitus, or malignancy [5, 6]. While the pathophysiology remains unclear, histological analysis appears to suggest a chronic progression of fat necrosis to inflammation to fibrosis, resultant from an initial proinflammatory cytokine response [7].

Management of SM and treatment regimens is based on case reports and other fibrosing diseases, such as sclerosing encapsulating peritonitis [8]. The most commonly cited treatment regimen includes the combination of 40 mg daily prednisone and 10 mg twice daily tamoxifen [1]. This is continued for 3 months, followed by a gradual prednisone taper if there is symptomatic improvement. Tamoxifen is recommended to continue indefinitely due to the high rate of relapse seen in other fibrotic diseases when tamoxifen is discontinued [1]. Other agents have been explored as well, including the angiogenesis inhibitor thalidomide. One small, open-label pilot study looked at 5 patients with symptomatic SM who were treated with 200 mg thalidomide nightly for 3 months, with 4 reporting clinical response and 1 complete remission. As thalidomide is known for adverse effects including peripheral neuropathy and teratogenicity, it is recommended only in those who either failed or who have contraindications to corticosteroid/tamoxifen therapy [9].

There are a few case reports written about successful SM treatment with TNF-alpha inhibitors, such as infliximab and adalimumab. Rothlein et al. described a case report about a patient with sacroiliitis and similar presentation of SM with debilitating paroxysmal abdominal pain, who was successfully treated with significant improvement in abdominal symptoms with infliximab treatment [10]. In another study, Nyberg et al. presented 27 patients with SM. In this group, for one patient, despite therapy with prednisone and methotrexate, treatment was initiated with the TNF-alpha inhibitor adalimumab for a psoriatic arthritis flare, which resulted in decreased abdominal pain [11].

Sharma et al. in a systematic review of 192 patients with SM reported that, in terms of medical treatment, the majority of patients (*n* = 56, 83.5%) received steroids. TNF-alpha inhibitor, infliximab, was given to only 2.9% of participants (*n* = 2) with other treatment options reported as well—colchicine, tamoxifen, 6-mercaptopurine, antibiotics, azathioprine, methotrexate, cyclophosphamide, IVIG, and D-penicillamine. Since there was an overlap between the various agents of medical treatment, it was not possible to address their efficacy in this particular study but again showed a potential role for biologic therapy in SM [3].

The role of pentoxifylline therapy in SM is also not yet well established. Kapsoritakis et al. described a case report of retractile mesenteritis with malabsorption syndrome, which was successfully treated with pentoxifylline therapy (800 to 1200 mg/day). An exact mechanism of pentoxifylline antifibrotic/anti-inflammatory activity is unknown. In some studies, it is reported that pentoxifylline could act on the synthesis of proinflammatory cytokines—TNF-*α*, intercellular adhesion molecule-1, IL-1*β*, IL-6— and activation of lymphocytes [12].

Surgical therapy is indicated for those who do not respond to medical management and have evidence of persistent mechanical obstruction or bowel ischemia. Specific procedures performed vary according to involvement of the mesentery and degree of obstruction or ischemia and can include bowel resection, mass resection, or palliative bypass [1].

## 4. Conclusion

SM is a rare disease, and therefore sufficient data are unavailable regarding clinical course and treatment. For this reason, it is vital that physicians have a broad differential diagnosis when patients come with abdominal pain into the emergency room. Since a gold standard treatment of patients with SM has not yet been clearly established, our clinical case of steroid/tamoxifen-resistant SM with a good response to TNF-alpha inhibitors/pentoxifylline may represent an important clinical interest for consideration in future patients.
